# How do fluctuating ecological dynamics impact the evolution of hosts and parasites?

**DOI:** 10.1098/rstb.2022.0006

**Published:** 2023-03-27

**Authors:** A. Best, B. Ashby

**Affiliations:** ^1^ School of Mathematics and Statistics, University of Sheffield, Sheffield S3 7RH, UK; ^2^ Integrative Biology, University of California - Berkeley, Berkeley, CA 94720-5800, USA; ^3^ Department of Mathematics, Simon Fraser University, Burnaby, British Columbia, Canada V5A 1S6; ^4^ Department of Mathematics, University of Bath, Bath BA2 7AY, UK

**Keywords:** evolution, host–parasite, adaptive dynamics, eco-evolutionary dynamics

## Abstract

Theoretical models of the evolution of parasites and their hosts have shaped our understanding of infectious disease dynamics for over 40 years. Many theoretical models assume that the underlying ecological dynamics are at equilibrium or constant, yet we know that in a great many systems there are fluctuations in the ecological dynamics owing to a variety of intrinsic or extrinsic factors. Here, we discuss the challenges presented when modelling evolution in systems with fluctuating ecological dynamics and summarize the main approaches that have been developed to study host–parasite evolution in such systems. We provide an in-depth guide to one of the methods by applying it to two worked examples of host evolution that have not previously been studied in the literature: when cycles occur owing to seasonal forcing in competition, and when the presence of a free-living parasite causes cycles, with accompanying interactive Python code provided. We review the findings of studies that have explored host–parasite evolution when ecological dynamics fluctuate, and point to areas of future research. Throughout we stress the importance of feedbacks between the ecological and evolutionary dynamics in driving the outcomes of infectious disease systems.

This article is part of the theme issue ‘Infectious disease ecology and evolution in a changing world’.

## 1. Introduction

There is now a vast literature of theory on the evolution of hosts and their parasites [1,2]. A wide range of questions about the evolutionary dynamics of host–parasite relationships have been explored, including the nature and role of infection genetics [3–10], the distinction between host tolerance and resistance [11–16], the impacts of spatial structure [17–24], the effect of predation [25–29], the impacts of co-infection and superinfection [30–35] and more besides. The study of fluctuating dynamics in the host–parasite literature has primarily focused on either epidemiological cycles [36–38] or fluctuating selection in the context of host–parasite coevolution (e.g. so-called ‘Red Queen dynamics’ induced by negative frequency-dependent selection; [39]). However, few studies have considered how fluctuating ecological dynamics affect host or parasite evolution. Yet the ecological world is not constant. Whether owing to extrinsic factors such as seasonality or intrinsic factors such as time lags, ecological dynamics may fluctuate over time [40]. In particular, variation in population sizes is likely to affect contact rates between hosts and parasites, and hence the strength of selection for traits such as resistance and virulence [41]. It is, therefore, important to understand how fluctuating population sizes impact selection on host and parasite traits.

Many theoretical models assume that host and parasite population sizes are constant or infinite or that the population dynamics are uncoupled from evolutionary dynamics (see [41]), in which case fluctuating population dynamics either are prohibited by model design or are assumed to have no impact on selection. ‘Eco-evolutionary’ models, on the other hand, incorporate population dynamics from the outset and therefore naturally capture feedbacks between ecological and evolutionary processes, which may or may not feature fluctuations in population sizes. Population dynamics can play a major role in host and parasite evolution, with several recent studies showing how feedbacks between ecological and evolutionary processes cause qualitative shifts in evolutionary outcomes [41–44]. However, the effects of fluctuating population dynamics on evolution are rarely studied in these systems. In particular, while models of host–parasite coevolution often exhibit fluctuating ecological dynamics, most studies instead focus on fluctuations in allele frequencies or in trait values.

To capture the effects of eco-evolutionary feedbacks, which invariably complicate matters, theoreticians often use techniques such as evolutionary invasion analysis, also known as ‘adaptive dynamics’ [45–48], which make simplifying assumptions about the underlying genetics (i.e. quantitative traits) and mutational process (i.e. mutations are rare with small phenotypic effects) to facilitate model analysis (this is of course just one modelling approach and alternative frameworks can also be used). In practice, the adaptive dynamics approach requires a separation of timescales between ecological and evolutionary dynamics, while still maintaining critical feedbacks between these processes, which means that we only need to consider the invasion fitness of a rare mutant in a resident population at its ‘dynamic attractor’. In other words, one assumes that the ecological dynamics of the resident population settle into their long-term pattern of behaviour before a new mutant arises. In most studies, the resident population tends to a stable equilibrium, which conveniently makes the invasion analysis relatively straightforward (see below). While non-equilibrium population dynamics in host–parasite systems are rarer, they can be generated by diverse factors, including free-living parasite stages [36], parasitic castration [17], seasonality [49], time lags [50] and stochasticity [51]. The techniques for analysing models with non-equilibrium population dynamics are more complicated, and therefore few host–parasite models in the literature consider scenarios that lead to non-equilibrium population dynamics [52–60].

Here, we focus on ‘deterministic fluctuations’, or more mathematically speaking, limit cycles, in ecological dynamics induced by extrinsic or intrinsic factors. Some of the approaches we discuss would be equally applicable to chaotic and/or discrete fluctuations, but more likely these may require alternative methods [61,62]. We begin by outlining why modelling evolution with fluctuating population dynamics is challenging, and then discuss possible modelling approaches to overcome these challenges. We then examine two previously unstudied worked examples of how we could model host evolution in fluctuating environments. Our two novel applications are (i) when fluctuations occur owing to seasonally varying resources (as opposed to seasonally varying births used in previous models [54,55]) and (ii) when fluctuations occur intrinsically owing to free-living parasite stages rather than to external forcing. We then summarize the existing literature on host–parasite evolution with fluctuating population dynamics, and finish by discussing possible future directions for research in this area.

## 2. Why is it challenging to model evolution with fluctuating ecological dynamics?

To answer this question let us consider a relatively simple model of host defence evolution. The dynamics of resident susceptible (*S*) and infected (*I*) hosts are given by the following ordinary differential equations (ODEs),

2.1 $$\frac{dS}{dt}=(a-q(S+I))S-bS-\beta SI+\gamma I$$

and

2.2 $$\frac{dI}{dt}=\beta SI-(b+\alpha +\gamma )I.$$

Susceptible hosts reproduce at rate *a*, with a reduction owing to crowding by *q*. All hosts die at natural mortality rate *b*, while infected hosts have additional mortality caused by parasite virulence at rate *α*. Infection is a density-dependent process with parameter *β* and infected hosts can recover to being susceptible again at rate *γ*. In this system, provided the parasite’s basic reproductive ratio, *R*_0_ = *βS*_dfe_/(*b* + *α* + *γ*) > 1, where *S*_dfe_ is the disease-free equilibrium, the resident population reaches an equilibrium—a key point to remember—with the equilibrium densities given by

$$S^{*}=\frac{b+\alpha +\gamma }{\beta },\;I^{*}=\frac{(a-qS^{*}-b)S^{*}}{(q+\beta )S^{*}+\gamma }.$$

We will apply the framework of adaptive dynamics to model evolution [45–48]. We assume a rare mutant host arises which has a small difference in the transmission rate, with lower *β* meaning a better defended host (owing to decreased susceptibility to infection). We will also assume that there is a cost to defence through a lowered reproduction rate, such that *a* = *a*(*β*). Given that the mutant is rare we can assume mutant–mutant interactions do not impact its dynamics at early time points. Let us initially take the simplifying assumption that there is no recovery, i.e. *γ* = 0. This means the mutant’s initial dynamics can be given by

2.3 $$\frac{dS_{m}}{dt}=(a(\beta_{m})-q(S^{*}+I^{*}))S_{m}-bS_{m}-\beta_{m}S_{m}I^{*}.$$

In this simple example where we assumed *γ* = 0, infected hosts make no direct contribution to fitness, and the invasion fitness is simply the exponential growth rate of mutant susceptible hosts, that is,

2.4 $$s(\beta_{m},\beta )=a(\beta_{m})-q(S^{*}+I^{*})-b-\beta_{m}I^{*}.$$

Since all parameter values here are constants, S* and I* are equilibria, and *a*(*β*) is some specific function, this yields a simple numeric value for any value *β*_m_, and thus the fitness of an invading mutant can be easily determined. If *s*(*β*_m_, *β*) > *s*(*β*, *β*), then the mutant can invade.

In the case where *γ* > 0 the mutant host fitness is no longer simply the exponential growth rate of susceptible hosts since infected hosts are directly contributing to fitness. In this case, the fitness would be given by the dominant eigenvalue of the mutant’s part of the system at the resident–mutant equilibrium. In practice this fitness can be found through a number of methods, including direct determination of the eigenvalues, the next-generation matrix [63] or a sign-equivalent proxy by finding the determinant of the mutant’s part of the system [64]. For example, by the next-generation method the fitness in our model when *γ* > 0 would be,

2.5 $$s(\beta_{m},\beta )=\frac{a(\beta_{m})-q(S^{*}+I^{*})}{b+\beta_{m}I^{*}}+\frac{\gamma \beta I^{*}}{\left(b+\beta_{m}I^{*})(\alpha +b+\gamma \right)}-1.$$

While more complicated than the case where there is no recovery (*γ* = 0), all values in this expression—including the densities S* and I* are still constants, yielding a simple numeric value and a straightforward criterion for invasion: *s*(*β*_m_, *β*) > *s*(*β*, *β*). Given that a strain invading itself will have 0 fitness, this equates to *s*(*β*_m_, *β*) > 0.

**Figure 1.**
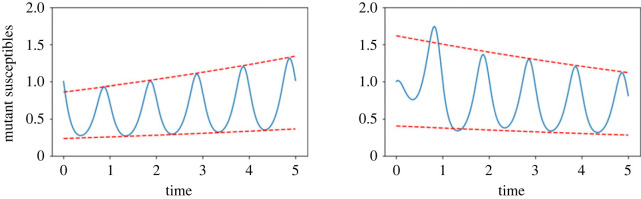
Example early time mutant trajectories, with envelopes created by the Floquet multipliers plotted as dashed lines. Example dynamics of mutant susceptible densities from the first example model, equations (3.1)–(3.3).

Let us now assume that instead of an equilibrium, the resident ecological dynamics reach a stable cycle. This may be due to intrinsic cycles in the system or due to extrinsic ‘seasonal forcing’ of parameters. For example, let us take our model above but where we assume the birth rate fluctuates over the course of a year, with

$$a(\beta ,t)=a_{0}(\beta )\times (1+\delta \sin(2\pi t)),$$

where *a*_0_ is the average birth rate (and is still involved in a trade-off with *β*), *δ* ∈ [0, 1] is the amplitude of the oscillations and the term 2*πt* ensures a period of 1 year. Now the resident populations are no longer at equilibrium, but will vary depending on the time point. This means we can no longer substitute a single value into our expressions for invasion fitness above. In the previous scenario, the timing of a mutation did not matter as the resident population was assumed to be at equilibrium. But if the resident population densities fluctuate, then the invasion fitness will also fluctuate, and so a mutant may be more fit than the resident at certain time points, and less fit at others. This point is demonstrated in figure 1, where the early time dynamics of the mutant densities are plotted, in the first case for an ultimately successful mutant and in the second for one that fails to invade. In both cases, however, we see that the mutant density may be higher or lower than its starting value (meaning a point estimate of the density is unreliable as a fitness measure) and moreover the densities may be increasing or decreasing depending on when the densities are examined (meaning a point estimate of the gradient is also unreliable as a fitness measure). How, then, can we handle situations where the population densities are time-dependent, and hence the timing of a mutation matters? A number of methods have been used, which we summarize below.

### (a) Running numerical simulations

A relatively simple approach is to run numerical simulations of the evolutionary process. A multi-strain system is established with initially only one strain present. The dynamics are then run using numerical ODE solvers for some fixed time. At this point any strains below some threshold density are taken as extinct, and a mutant strain is chosen to be adjacent to the current dominant resident. This routine is then repeated multiple times, and by recording all strains present at each mutation step the evolutionary trajectory can be visualized. This approach is used in a number of studies of standard models as a visual confirmation of the analytic results (for example [64,65]). Alternatively, a stochastic simulation algorithm could be used where numerical ODE solvers are not required and the mutation rate is a parameter of the model. Such simulation approaches are relatively simple since there is no need to derive any expressions for fitness. Of course, this itself comes at a cost to understanding of the outcomes. Moreover, such simulations may take some time to reach an evolutionary attractor, as the nature of the cycles may mean that what should be a dominant strain happens to have a low density at the mutation step and is thus made extinct.

### (b) Deriving the fitness algebraically

The formal groundwork for considering evolution in variable environments was laid by Metz *et al.* [62], who noted that the fitness of a mutant in a variable environment is given by its dominant Lyapunov exponent. For a system where the attractor of the population dynamics is an equilibrium, this is simply the largest eigenvalue. However, for a system with regular cycles, as we have discussed, this is more complex.

If the population is unstructured, and so the mutant dynamics are given by a single ODE, we can still calculate the fitness as being the average growth rate of a rare mutant over one cycle period. That is, we write d*X*_m_/d*t* = *r*(*t*)*X*_m_, and find the expression for *r*(*t*). For our example system, if there were no recovery and the cycles run from time *P*_0_ to *P*_1_ with period *T*, we could, therefore, write

2.6 $$r=\frac{1}{T}\int_{P_{0}}^{P_{1}}r(t)dt=\frac{1}{T}\int_{P_{0}}^{P_{1}}(a(\beta_{m},t)-q(S^{*}(t)+I^{*}(t))-\beta_{m}I^{*}(t))dt-b.$$

In some cases, we can obtain algebraic expressions for all of the time-dependent variables and parameters, allowing us to continue with a full evolutionary analysis much as we would have in an equilibrium system. Often, however, the integral of the variables cannot be simply expressed, and we would need to numerically calculate those values for a given parameter set. Studies taking this approach in host–parasite systems include Donnelly *et al.* [53] and Hite & Cressler [57].

### (c) Deriving the fitness numerically

Generally, host–parasite systems are structured, however, and the mutant’s growth rate is not a simple linearized expression. In these cases, we can instead use a numerical routine to calculate the Lyapunov exponents, which for fluctuating systems are generally referred to as ‘Floquet exponents’. In vector notation, if **X**(*t*) = (*S*_m_(*t*), *I*_m_(*t*)) and the period of the cycles is *T*, then the early time dynamics can be given by

$$X(t+T)=P(t)\;e^{\mu_{i}T}.$$

Since **P**(*t*) is a periodic function, whether the population grows or shrinks ultimately depends on the values of *μ*_*i*_, which are the Floquet exponents. We can think of the term $e^{\mu_{i}T}$ (the Floquet multiplier) as creating an envelope from which the dynamics cannot escape. Therefore, if *μ*_*i*_ < 0 for all *i*, the envelope shrinks asymptotically towards zero and so must the mutant densities. By contrast, if *μ*_*i*_ > 0 for any *i* then the envelope grows and the density will grow asymptotically. Examples of this can be seen in figure 1, with the Floquet multipliers shown as the dashed lines creating the envelopes that indicate the overall trajectory (after some initial transitory behaviour), even though there are short time periods where the densities are in the opposite direction—causing the problems with simulation approaches outlined above.

This leaves the question of how we go about finding the values of *μ*. As we show below using two worked examples, we write our system as

X(t+T)=X(t)C,

calculating the values of the matrix **C** through a numerical routine. The eigenvalues of this matrix are $\rho_{i}=e^{\mu_{i}T}$, and we can, therefore, calculate the values of *μ*_*i*_. This approach has been applied to a different ecological model by Klausmeier [66] and to host–parasite systems by Ferris & Best [54,55] and Ferris *et al.* [56].

### (d) Deriving an approximate selection gradient

In recent work, Lion & Gandon [59] built on methods from constant environments where contributions to fitness are calculated as a product of a mutant’s quantity and its quality [67,68]. In a fluctuating environment, this selection gradient is again averaged over one period of a cycle, similarly to calculating the Floquet exponent directly as above. Using this method, while the resulting expression is only approximate—in particular requiring evolutionary and convergence stability to be numerically checked separately—we can gain a biologically meaningful expression for the fitness even in a structured population.

## 3. Two worked examples

Here, we will demonstrate the method developed by Ferris & Best [54] with two worked examples that have not previously been examined in the literature. In both cases we will consider the evolution of host avoidance of parasitism (i.e. lowered transmission rates) at a cost to reproduction. The underlying epidemiological model is as given above. Python code to accompany both examples is available as a fully functional, interactive Jupyter Notebook (https://mybinder.org/v2/gh/abestshef/fluctuating/HEAD?labpath=evo_flux.ipynb) and can also be downloaded from GitHub (https://github.com/abestshef/fluctuating).

### (a) Example 1. Seasonally varying resources

In our first case, we assume the amount of resources available to hosts varies seasonally over the course of a year. This is incorporated into the model by making the competition term, *q*, a sinusoidal function of time, completing one cycle each year. Our epidemiological model could then be updated to

3.1 $$\frac{dS}{dt}=(a-q(t)(S+I))S-bS-\beta SI+\gamma I$$

and

3.2 $$\frac{dI}{dt}=\beta SI-(b+\alpha +\gamma )I$$

with *q*(*t*) = *q*_0_(1 + *δ*sin(2*πt*)). The amplitude of the variation, i.e. the ‘size’ of the effect is given by *δ* ∈ [0, 1].

Considering the growth of a rare mutant host type, and using the 'next generation' approach as outlined above, the fitness of the mutant can be given by

3.3 $$s(\beta_{m},\beta )=\frac{a(\beta_{m})-q(t)(S^{*}(t)+I^{*}(t))}{b+\beta_{m}I^{*}(t)}+\frac{\gamma \beta I^{*}(t)}{\left(b+\beta_{m}I^{*}(t))(\alpha +b+\gamma \right)}-1,$$

where S*(*t*), I*(*t*) represent the stable limit cycles of the resident populations. Given the period of our varying function is 1 year we may well expect the dynamics to vary yearly as well. However, it is well known that such systems can give rise to period-doubling bifurcations, leading to multi-annual cycles (and even chaotic dynamics), so the period should be checked. We find in this model only annual cycles occur for the parameter ranges presented.

How can we gain a measure of host fitness in this case given that the population densities are never at equilibrium? Thankfully we have centuries of mathematical theory to rely on, principally that known as Floquet theory. The method developed by Ferris & Best [54] takes advantage of these classic results. In particular we can find the Floquet exponent as follows:

1. Run the resident dynamics using a numerical solver for ODEs for such time that they have reached their dynamic attractor, i.e. their annual limit cycle in this case.
  (a) A useful trick to speed up this step when looping through parameter values is to set the initial condition as the final value of the previous run.
  (b) Seasonal models are often ‘stiff’, where the dynamics follow two very different timescales. In these cases, standard numerical ODE solvers tend to perform poorly. A solution is to use a numerical solver built for such stiff systems (for example in the accompanying Python code we use the optional argument, method=‘Randau’).
2. Run two numerical simulations of the resident–mutant dynamics—with an assumption of rare mutants—for just a single cycle period (or multiple periods if it is known that period-doubling may occur). The two runs should have initial conditions for the residents given by the last values found in step 1; for the mutants the two runs should have ‘linearly independent’ initial conditions, and we can simply take [0,1] and [1,0] for ease.
3. Form the square matrix **C** that consists of the values of the mutant *S*_m_ and *I*_m_ densities at the end of the runs in step 2.
4. Calculate the largest eigenvalue of **C**, and take its natural logarithm to find the value of the Floquet exponent, and hence the fitness.

This approach allows us to compute pairwise invasion plots (PIPs), and/or further numerical routines to find the local fitness gradient, and therefore find the location and nature of singular strategies.

**Figure 2.**
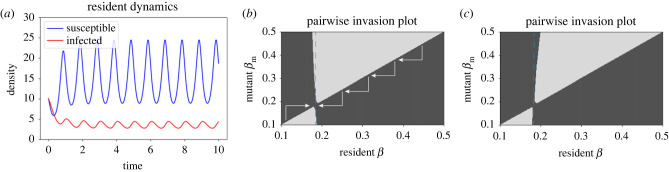
Resident dynamics of the system with seasonally varying resources (equations (3.1) and (3.2)), and two pairwise invasion plots. In (*a*), *a* = 10 and *β* = 0.2. In (*b*,*c*), we take a trade-off given by $a=10-(\tau_{1}^{2}/\tau_{2})(1-\exp((\beta -0.2)\tau_{2}/\tau_{1}))$ with *τ*_1_ = 1.58, which would give a singular point at *a* = 10, *β* = 0.2 in the non-seasonal model. In (*b*), *τ*_2_ = −3 and in (*c*) *τ*_2_ = 3, which alter the curvature of the trade-off, and hence alter the stability conditions from a continuously stable strategy (CSS) in (*b*) to an evolutionary branching point in (*c*). Default parameter values: *b* = 1, *α* = 1, *γ* = 1, *q*_0_ = 0.5, *δ* = 0.5. (Online version in colour.)

Figure 2 shows the resident dynamics for a fixed parameter set and two example PIPs. PIPs are a commonly used plot in adaptive dynamics, with the colours denoting whether a mutant–resident pair results in the mutant invading (black) or not (grey) [46]. Through small evolutionary steps the population will evolve up or down the main diagonal, as shown by the arrows, until a singular point is reached where there is a crossing point. In this first case, the dashed vertical line through the singular point lies entirely in a region of negative fitness, meaning that strategy cannot be invaded. As it is both attracting (convergence stable) and uninvadable (evolutionarily stable) we call this a continuously stable strategy (CSS). In the second PIP, we identify the potential for evolutionary branching, where the population is attracted to the singular point (convergence stable), but once there any other mutant can invade (evolutionarily unstable). This means the population will undergo disruptive selection and branch into two coexisting resident types [46]. While this is a known result for host resistance evolution in standard models, especially when trade-offs are weakly decelerating [69], it is notable that it remains in a system with fluctuating densities.

**Figure 3.**
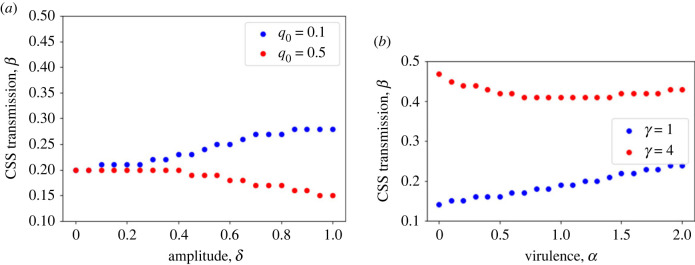
Location of continuously stable strategies (CSSs) in the model for seasonally varying resources as (*a*) the amplitude of forcing, *δ*, and competition, *q*_0_, and (*b*) virulence, *α*, and recovery, *γ*, are varied. Increasing the amplitude of forcing causes the CSS to increase when competition is relatively weak (*q*_0_ = 0.1) but has the opposite effect when competition is relatively strong (*q*_0_ = 0.5). Default parameter values are as in figure 2. (Online version in colour.)

In figure 3, we focus on how optimal investment in avoidance at a CSS varies with model parameters. First we directly assess the impact of seasonality by varying the amplitude of the oscillations. In figure 3*a*, we clearly see the effect of introducing fluctuations, as the location of the CSS varies substantially when there are large-amplitude seasonal oscillations compared with when the amplitude is 0 (i.e. no seasonality). Moreover, we see that the direction in which the CSS changes depends on the level of competition, with high amplitudes leading to higher transmission (lower resistance) when baseline competition is low, but lower transmission (higher resistance) if baseline competition is high. Why does this result arise? The answer can be found by exploring the population dynamics as both competition and amplitude vary, and considering how these might affect selection. If baseline competition is low (e.g. *q*_0_ = 0.1), infected densities are higher than if competition is high (e.g. *q*_0_ = 0.5). Increasing the amplitude of oscillations increases both the average and maximum infected densities over a cycle. For low competition, this leads to extremely high infected densities at large amplitudes, meaning infection becomes almost inevitable for a host, limiting the benefit of evolving costly resistance. As such, the CSS shifts to higher transmission and births (since infected hosts recover, infected hosts may yet be able to contribute to reproduction at a later point). By contrast, for high competition, even at high amplitudes the average and maximum infected densities are not too large (in fact now the susceptible densities become much larger). In this case, the relatively small increase in infection with increasing amplitude is worth mitigating by evolving lowered transmission, and the high susceptible densities mean the effect on overall reproduction is not too large.

In figure 3*b*, we do not directly examine the effect of oscillations *per se*, but instead examine whether a well-known result from non-fluctuating models—that resistance is maximized at lowest virulence when there is no recovery, but at intermediate virulence when there is—is maintained when oscillations are introduced. We see that increased virulence selects for higher transmission (lower resistance) when there is little recovery, but that higher recovery rates lead to a ‘U-shaped’ investment with virulence, in accordance with non-seasonal models [70,71]. This is because if hosts can return to being susceptible—and therefore to reproduce—selection to avoid infection is weakened.

### (b) Example 2. Free-living parasite stages

In our second case, we do not extrinsically ‘force’ fluctuations on the system, but instead note that in certain model formulations limit cycles intrinsically arise as an outcome. In host–parasite systems, a well-known example of this is when there are free-living parasite stages that drive transmission. Such a population could be modelled as follows:

3.4 $$\frac{dS}{dt}=(a-q(S+I))S-bS-\beta SP+\gamma I,$$

3.5 $$\frac{dI}{dt}=\beta SP-(b+\alpha +\gamma )I$$

3.6 $$\mathrm{and}\;\frac{dP}{dt}=\theta I-\delta P.$$

Now infection is not through direct contact of susceptible and infected individuals but through susceptible hosts picking up free-living parasite stages. There are many ways the dynamics of the free-living stages can be modelled; here we assume that stages are shed at a constant rate, *θ*, by infected hosts, that these stages decay at rate *δ* and that loss of these stages due to infection is negligible. The dynamics of this system can be both equilibria and cycles depending on the parameter values.

It is common for the dynamics of this system to be sufficiently stiff that even the specialist numerical ODE solvers struggle to run for long time periods. In this case, we recommend log-transforming the model. This involves taking new variables, *X* = ln(*S*), *Y* = ln(*I*) and *Z* = ln(*P*). This leads to a transformed model given by

3.7 $$\frac{dX}{dt}=a-q(\;e^{X}+\;e^{Y})-b-\beta \;e^{Z}+\gamma \;e^{Y-X}$$

3.8 $$\frac{dY}{dt}=\beta \;e^{X+Z-Y}-(b+\alpha +\gamma )$$

3.9 $$\mathrm{and}\;\frac{dZ}{dt}=\theta \;e^{Y-Z}-\delta .$$

**Figure 4.**
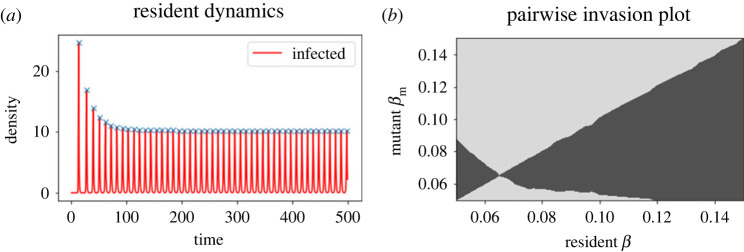
Resident dynamics and pairwise invasion plot (PIP) for the system with a free-living parasite (equations (3.7)–(3.9)). Note that since this model has intrinsic cycles (as opposed to the externally forced model in figure 3), there is no comparison to be made between cycling and non-cycling versions of the model. In (*a*), *a* = 10 and *β* = 0.1. In (*b*), we take a trade-off given by $a=10-(\tau_{1}^{2}/\tau_{2})(1-\exp((\beta -0.1)\tau_{2}/\tau_{1}))$, with *τ*_1_ = 75 and *τ*_2_ = −400. Default parameter values: *b* = 1, *α* = 1, *γ* = 0.1, *q*_0_ = 0.1, *μ* = 0.1, *θ* = 5. (Online version in colour.)

A simple reverse transformation of, e.g. *I* = e^*Y*^ can then be used to plot or record the densities as in figure 4*a*.

The fitness of a mutant host in this system looks relatively similar to above,

3.10 $$s(\beta_{m},\beta )=\frac{a-q(S^{*}(t)+I^{*}(t))}{b+\beta_{m}P^{*}(t)}+\frac{\gamma \beta P^{*}(t)}{\left(b+\beta_{m}P^{*}(t))(\alpha +b+\gamma \right)}-1.$$

While it is not explicit that there are fluctuations present, it is known that for a wide range of parameter space the attractor of the resident dynamics, and hence the densities S*(*t*), I*(*t*) and P*(*t*) are limit cycles. We, therefore, must again explore how to attain fitness in this case.

The method is in fact identical to that above, but with one added complication. Previously we could assume that the period of the fluctuations was the same as the forcing period (taken to be 1 above), or perhaps some simple multiple of it in case of period-doublings. In the case where there are intrinsic limit cycles it is unlikely to even be an integer value. While in simple models we can calculate the period explicitly from details of the model, in general we must add a stage between steps 1 and 2 above to calculate the period numerically. This can be done by finding peaks at later time points in the resident dynamics and calculating the time between them. Most programming languages have such a built-in function—in our accompanying Python code we use the ‘find_peaks’ function in the SciPy library. We can then continue as we did above. Figure 4 shows the resident dynamics, with the peaks identified by the find_peaks function picked out with ‘X’ marks. For the example used here we find that now the period of the dynamics is 10.13 time-units (to two decimal places). Moreover the PIP demonstrates that the method continues to work with the numerical estimate of the period.

**Figure 5.**
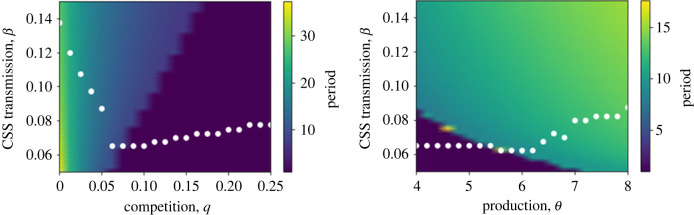
Plots of how the continuously stable strategies (white dots) vary in the model with a free-living parasite (equations (3.7)–(3.9)) as (*a*) competition, *q*, and (*b*) parasite production rate, *θ*, are varied. Heatmap colouring indicates the period of the oscillations. Parameter values are as described in figure 4. (Online version in colour.)

In figure 5, we highlight how the CSS investment changes as model parameters are varied. In this case, we show these CSS points against a colourmap of the period of the underlying cycles that are present. In both cases, it is notable that the trend of the CSS is different depending on whether the underlying population dynamics are cycles (lighter colours) or equilibria (dark-blue). For low levels of competition the dynamics are cycles, and the CSS transmission rate rapidly drops as competition increases, but once it moves to a region of equilibria the CSS transmission gradually increases. This effect is mirrored by varying the rate of parasite production; for low production levels the dynamics are equilibria and there is a slight downward trend, but as production increases cycles emerge and the CSS transmission increases. Why do we see these patterns? Focusing on competition, in the equilibrium region, increasing competition leads to higher transmission (lower resistance). Similarly to the high competition case in figure 3*a*, selection to avoid infection is greatest at low competition when the infected and parasite densities are highest, because the force of infection is never too high. However, when cycles emerge, the infected and parasite densities increase dramatically, especially at their maximum value on a cycle. This means that hosts are now facing extremely high probability of infection, and there becomes limited benefit to evolving costly resistance. These plots highlight how the existence of population cycles creates a fundamental, qualitative change to the evolutionary dynamics, highlighting that there is a two-way feedback between ecology and evolution.

## 4. What have existing theoretical models told us?

To our knowledge, the first theoretical study to consider the evolutionary impact of ecological oscillations in a host–parasite system was Koelle *et al.* [58]. They included a seasonal driver causing annual fluctuations in transmission, but assuming a constant population size. The parasite was assumed to have a trade-off between sensitivity to this climate driver and maximum transmission. Primarily using numerical simulations, they found that increased climate variability would lead to parasites evolving reduced sensitivity to those fluctuations.

Following this a number of studies have examined parasite evolution with fluctuating ecological dynamics. Sorrell *et al.* [60] included a seasonally forced reproduction rate in a study of covert parasitism with superinfection, where covertly infected hosts do not transmit horizontally (but can transmit vertically) but can become overt at a later stage. They found that when the amplitude of seasonality is small there is no selection for covert infections, but once a threshold is passed a degree of covert infection will be selected for. This is because covertly infected hosts survive longer than those overtly infected (as they do not suffer from virulence), creating a reservoir of infection that better copes with the drops in population densities during a cycle. Donnelly *et al.* [53] explored a more classic transmission–virulence trade-off with seasonally forced host reproduction. Under standard assumptions the parasite fitness could be found analytically (since the average host density over one period is constant), and they found that parasite fitness was in fact unaffected by the amplitude or period of the seasonal forcing. However, if virulence is density-dependent then parasite fitness depends on the average total population density, which is not constant over a period. Numerically calculating the average densities and substituting into the fitness, they found parasites were selected to evolve higher virulence and infectivity at higher amplitudes of seasonality as increased amplitude with density-dependent virulence leads to lower susceptible densities, requiring greater exploitation by the parasite to survive. Hite & Cressler [57] also examined a classic transmisison–virulence trade-off, but where host growth depends directly on resources, with fluctuations emerging intrinsically. They found that in regions where fluctuations occurred, there can be evolutionary bistability such that the parasite is driven to either extremely high or extremely low levels of virulence, but that when the high-virulence type occurred it partially stabilized the cyclic dynamics.

Recently, Lion & Gandon [59] applied their approach (see §2) to three case studies of parasite evolution. They found (i) unlike in constant environments, longer-lived parasites can become more virulent in fluctuating environments, (ii) pathogens can evolve a preference for hosts more damaged by infection, in opposition to standard results in constant environments, and (iii) fluctuations reduce selection for more virulent parasites in the presence of imperfect vaccines.

Studies have also examined the evolution of host defences in variable environments. Best *et al.* [52] examined the evolution of immune priming, for which the underlying population dynamics can exhibit intrinsic limit cycles in the absence of seasonal forcing. Using a numerical approach (somewhat more ad hoc than the method discussed above) they showed how hosts may evolve from equilibrium dynamics to a CSS in a region of limit cycles, particularly when host lifespan and sterility of infected are high. Studies including seasonal reproduction in classic host defence evolution models were then developed, through first avoidance (lowered transmission; [54]) and then tolerance (lowered mortality; [55]). In these studies, the formal numerical routine described above was developed. These studies showed that increased amplitude selects for lower avoidance but higher tolerance owing to the change in infected densities as amplitude increases, and how evolution towards a CSS may cause hosts to evolve through different underlying population dynamic regimes, for example from period-1 to period-2 cycles. Most recently, Ferris *et al.* [56] developed the first coevolutionary invasion analysis of a host–parasite model with fluctuating ecological dynamics, finding that when growth rates are parameterized by experimental data, both host defence and parasite virulence evolve to the highest levels at intermediate amplitudes of fluctuations owing to non-monotonic changes in host birth rate under the experimental conditions.

While our focus is on evolution in host–parasite systems, models of evolution with fluctuating ecological dynamics have been studied in other ecological scenarios. Notably these include models of preadator–prey systems [72–74] and discrete-time models of intraspecific competition [61,75].

## 5. Key trends and future questions

While there are relatively few studies in this area, there appear clear trends in results from models with fluctuating ecological dynamics. The first and most fundamental is that fluctuating ecological dynamics often significantly alter evolutionary outcomes. In host–parasite systems it has been shown that increased amplitude of seasonal forcing can lead to higher virulence in parasites [53] and lower avoidance in hosts [54], suggesting environments with greater fluctuations may be expected to lead to more prevalent, severe infections, though a full coevolutionary model would be required to confirm this.

These models also highlight the two-way feedbacks between ecological and evolutionary dynamics. For example, in their model of host defence, Ferris & Best [54] showed that increasing the amplitude of seasonal birth rate increases the infected density such that, when there are sufficient rates of recovery, hosts will be selected to lower their defence (as seen in our first model here). The combination of these effects can move the system from a region of period-1 cycles to a region of period-2 cycles, fundamentally altering the ecological environment of the host and parasite. Similarly, we have shown in this study how evolution can lead the system across the boundary between ecological equilibria and cycles. For parasite evolution, Sorrell *et al.* [60] showed that when environmental oscillations are small there could be no selection for covert parasite infections, but increasing the amplitude allowed covert infections to emerge, again substantially changing the ecological background.

There remain a raft of open questions to be considered using these methods. As already mentioned, one important direction is to explore coevolutionary dynamics in a fluctuating ecological environment. Previous coevolutionary models have highlighted how coevolutionary cycles can emerge without being driven by ecological cycles [42], yet the impact of *ecological* cycles on *coevolutionary* cycles has received relatively little attention [43,44]. In models of sexual versus asexual reproduction, coevolutionary cycles are often (but not always, e.g. [76]), crucial for the evolutionary maintenance of sex by parasitism. Hence, understanding how ecological cycles impact on coevolutionary cycles may shed new light on the Red Queen hypothesis for sex, which has been studied in depth for over 40 years [39,77]. Similarly, while examples of evolutionary branching when ecological dynamics are cycling have been found ([54], and herein), including one of the few examples of branching in host tolerance [55], it is not yet known whether ecological cycles increase or decrease diversification in general. Furthermore, it is unknown whether evolution differs when fluctuations are due to external forcing ([53–56,58–60], and our first model) or due to intrinsic factors ([52,57], and our second model). In particular, when cycles occur intrinsically we can compare the period and amplitude of the cycles with and without evolution to assess whether evolution is amplifying or suppressing ecological cycles.

While the basic tools discussed herein can be applied fairly broadly, an important methodological development will be to apply similar techniques to models with chaotic cycles, or more generally cycles without a fixed period. While numerical simulations of such systems could readily be carried out, any of the more formal techniques—including the one covered in detail in this study—require calculation of the eigenvalues, which currently requires integrating over a known time period of a cycle. While we can calculate the Lyapunov exponent for a chaotic system, placing this in the context of resident–invader dynamics is more challenging [61,62,78].

Exploring how the feedbacks between ecology and evolution impact host–parasite interactions remains a key direction for theoretical research. The work summarized in our study stresses this importance even further, showing that fluctuations in the ecological dynamics can alter selection on hosts and parasites, and that in turn the evolutionary trajectory can move the ecological dynamics of host–parasite systems into different qualitative as well as quantitative regimes. Much work in this area remains to be conducted, and this will give us much greater insight into a wide range of real biological systems.

## Data Availability

This article has no additional data.
